# Bacterial nitrogen-related phosphotransferase systems: new insights into diverse roles in virulence, physiology, and central metabolism

**DOI:** 10.1128/jb.00541-25

**Published:** 2026-03-23

**Authors:** Samalee Banerjee, Matthew T. Cabeen, Josephine R. Chandler

**Affiliations:** 1 Department of Molecular Biosciences, University of Kansas 4202, Lawrence, Kansas, USA; 2 Department of Microbiology and Molecular Genetics, Oklahoma State University 7618, Stillwater, Oklahoma, USA; University of California San Francisco, San Francisco, California, USA

**Keywords:** nitrogen-related PTS, nitrogen regulation, PTS, phosphotransfer, PtsN, PtsO, PtsP, HPr, EI, EII

## Abstract

Nitrogen-related phosphotransferase (PTS^Ntr^) systems are highly conserved and widely distributed in proteobacteria. These systems are thought to enable bacteria to sense and respond to changes in cellular carbon and nitrogen availability. The PTS^Ntr^ is analogous to the carbohydrate phosphotransferase systems that carry out phosphorylation and uptake of specific carbohydrates. Instead of targeting carbohydrates, the PTS^Ntr^ influences metabolic flux, virulence, biofilm development, stress adaptation, and other phenotypes in ways that vary among different species. Mechanistic insights into the regulatory roles of the PTS^Ntr^ have emerged from studies in diverse bacteria, including *Escherichia coli*, agriculturally relevant nitrogen-fixing symbionts, and biotechnology-relevant and opportunistic pathogens of the *Pseudomonas* genus. In this review, we summarize both seminal and more recent discoveries underpinning the current understanding of PTS^Ntr^ and identify unifying themes that connect these systems across disparate species.

## INTRODUCTION

The nitrogen-related phosphotransferase system (PTS^Ntr^) is found across almost all genera of proteobacteria, where it has demonstrated roles in virulence, central metabolism, and other physiological processes, such as biofilm formation. These systems are related to the more widely known carbohydrate phosphotransferase systems (PTS^Car^) but are structurally and functionally distinct. Some enzymes of the PTS^Ntr^ system are encoded within the same operon as the RpoN nitrogen-responsive sigma factor, which led to the initial naming of these systems as “nitrogen-related.” Studies of the PTS^Ntr^ in a wide variety of bacteria have contributed to an increasingly detailed molecular and functional understanding of these systems. Support for the “nitrogen-related” nature of these systems is provided by evidence that elements of the PTS^Ntr^ can directly sense and respond to nitrogen-containing byproducts of the TCA cycle (1, 2). The current understanding of these systems is that their primary function is to sense and respond to changes in cellular nitrogen and carbon levels and subsequently regulate metabolic and other processes related to nutrient optimization.

In the sections below, we will first provide a historical overview of the PTS^Ntr^, from the first report in 1989 to the later discoveries that have shaped our current understanding. Many of the seminal studies were carried out in *Escherichia coli*, several *Pseudomonas* species, and plant-root symbionts such as *Rhizobium*. In these and other species where it has been studied, PTS^Ntr^ can have very diverse roles but also share emerging common themes; many of these systems have crucial roles in virulence, metabolic and nutrient homeostasis, and other processes that appear related to balancing the use of nitrogen and carbon. This review aims to connect common regulatory themes across different bacteria, summarize the supporting evidence for the role of these systems in nitrogen-related regulatory responses, and describe how studies of the PTS^Ntr^ offer potential avenues for advancing the development of novel therapeutics.

## HISTORICAL OVERVIEW

### Initial discovery

The first report relating to the PTS^Ntr^ was in a 1989 study by Merrick and Coppard (1) that explored the effects of mutations in the open reading frames downstream of *rpoN*, encoding the nitrogen-responsive σ^54^ factor. They showed that a mutation in one of these open reading frames modulated transcription of the *nifH* gene, encoding a nitrogenase subunit, in *Klebsiella pneumoniae*. This open reading frame was later shown to encode PtsN, one of the three major PTS^Ntr^ enzymes (3). The role of this mutation in *nifH* regulation and the location within the *rpoN* operon led to the notion that PtsN-encoding gene had a role in nitrogen fixation or nitrogen-related metabolism.

**Fig 1:**
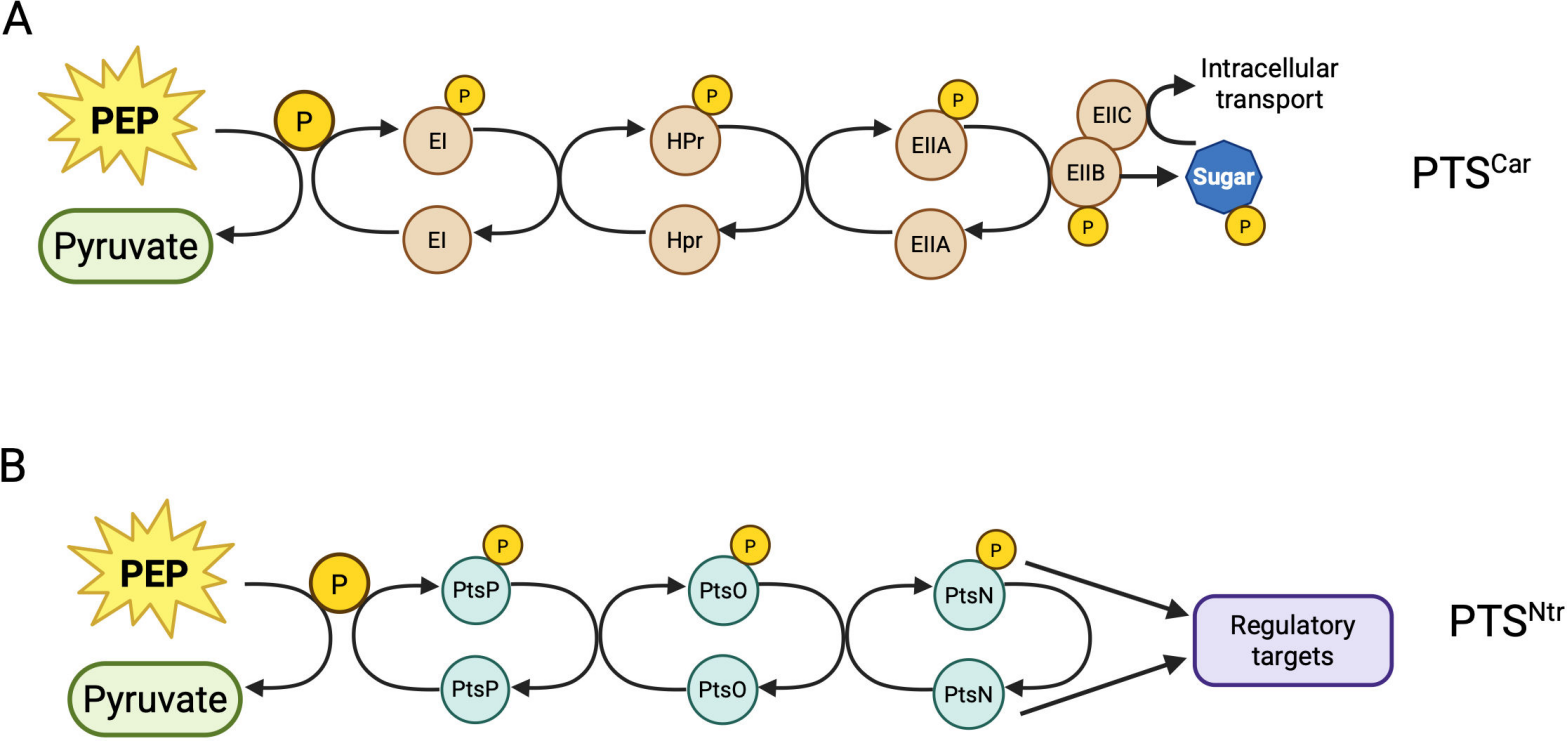
Illustration of carbohydrate (sugar)-related phosphotransferases (PTS^Car^, **A**) and nitrogen-related phosphotransferases (PTS^Ntr^, **B**). In both cases, the cascade begins with autophosphorylation of Enzyme I (EI in the carbohydrate-related PTS or PTS^Car^ and PtsP in the nitrogen-related PTS or PTS^Ntr^). This step transfers a phosphate from phosphoenolpyruvate (PEP). The phosphate is transferred from PtsP to an intermediate phosphocarrier enzyme, termed HPr in the PTS^Car^ and PtsO (or NPr) in the PTS^Ntr^, which then donates the phosphate to Enzyme II (PtsN in the PTS^Ntr^). In the PTS^Car^, the phosphate is further transferred to Enzyme II subunits or enzymes that then interact with and phosphorylate specific sugars. In the PTS^Ntr^, the phosphorylation state of PtsN controls its regulatory functions, allowing or prohibiting interactions with target proteins.

A few years later, Reizer et al. showed that PtsN was related to the group of enzymes in the fructose PTS called Enzyme IIA, often referred to as EIIA (3). EIIA is a key part of the PTS^Car^ that phosphorylates sugars and other substrates and transports them into the cell via a process termed group translocation. PTS^Car^ are found in both gram-positive and gram-negative bacteria; in contrast, PtsN is found only in proteobacteria. Fortunately, the existence of decades of work to understand the PTS^Car^ provided a mechanistic model for phosphotransfer within the PTS^Ntr^ as well as a convenient basis for comparison (for reviews of PTS^Car^, see references 4–6). The key similarity of PtsN proteins to PTS^Car^ was in the EIIA terminal phosphoacceptor domain. However, PtsN enzymes from several different species cohesively clustered into a distinct group from that of EIIA, suggesting the PtsN enzymes may be part of a functionally and distinctly different phosphorelay system (2). Reizer et al. (2) proposed that this newly discovered phosphorelay system might have nitrogen-related functions based on the prior findings by Merrick and Coppard (1). The subsequent naming of PTS^Ntr^ and PtsN was based on this assumption (7). Homologs of the other PTS^Car^ enzymes, now named PtsP (Enzyme I, or EI) and PtsO (HPr in PTS^Car^ and NPr in PTS^Ntr^), were subsequently identified by the same group and others (3, 7) (Fig. 1).

### PTS^Ntr^-dependent phenotypes and current view

The first few phenotypes associated with PTS^Ntr^ were related to nitrogen, although this association was not well understood at the time. Beyond the initial finding that *ptsN* mutations disrupted *nifH* regulation (1), a later finding by Powell et al. showed that *ptsN* mutants had growth defects on certain nitrogen sources (7). Other early studies described phenotypes with no association with nitrogen; for example, Powell et al. showed that *ptsN* mutation suppressed the lethality of a temperature-sensitive allele of *era* (7), a small G protein with a role in translation. Thus, the assumption that PTS^Ntr^ was actually nitrogen-related was tenuous at first, and it remained so for about a decade after its initial discovery. In fact, the entire naming convention of PTS^Ntr^ was questioned in a 2011 commentary (8)! However, these concerns were assuaged within the next couple of years.

In 2013, mechanistic studies of the PtsP protein in *E. coli* provided stronger evidence of a direct link of PTS^Ntr^ to nitrogen. These studies showed that PtsP interacts with and responds to the ligands glutamine and α-ketoglutarate (9). Glutamine and α-ketoglutarate are cellular signals for nitrogen and carbon abundance, respectively, such that their ratio indicates the relative availability of these two macronutrients. Based on these results, PtsP was proposed to sense the relative availability of cellular carbon and nitrogen. Importantly, glutamine-dependent regulation was later shown to be conserved in other species, including *Rhizobium leguminosarum* (10), *Sinorhizobium meliloti* (11), and *Caulobacter crescentus* (12), supporting the idea that sensing available cellular nitrogen is a conserved function of PTS^Ntr^. Although PTS^Ntr^ regulates different phenotypes in different bacteria, an emerging common theme is that these systems modulate different processes according to the cellular levels of carbon and nitrogen.

## GENERAL FEATURES

### PTS^Ntr^ components

The PTS^Ntr^ components are functionally analogous to the components of the PTS^Car^ (Fig. 1). Both PTS^Ntr^ and PTS^Car^ have three enzymes that are functionally related: PtsP/EI, PtsO (NPr)/HPr, and PtsN/EII for the PTS^Ntr^/PTS^Car^, respectively. In both systems, the phosphate is initially donated to PtsP/EI by phosphoenolpyruvate (PEP), a high-energy intermediate in glycolysis. Next, the phosphate is transferred from EI/PtsP to the phosphocarrier enzyme (PtsO/HPr). Finally, the phosphate is transferred to the terminal phosphoacceptor PtsN/EIIA. In all cases, phosphorylation occurs on conserved His residues (13). Importantly, phosphorylation is reversible, meaning that each enzyme can, in principle, phosphorylate or dephosphorylate its downstream enzyme (14–16).

PtsP, like EI, plays a key role in controlling the phosphorylation status of the downstream enzymes and, in particular, the terminal phosphoacceptor, PtsN or EIIA, which is the primary effector in both systems. In PTS^Car^, EIIA regulates phosphorylation of the sugar modifier EIIB. Unlike the three phosphoenzymes of both PTS systems, which are phosphorylated on His residues, the EIIB phosphorylation site is typically on a Cys residue. EIIB then phosphorylates the sugar substrate after it is transported through the membrane via the EIIC component (4). The PTS^Car^-related EIIA, EIIB, and EIIC enzymatic functions can all be part of the same protein or carried out by different proteins, depending on the system. Instead of a sugar substrate, the PTS^Ntr^ appears to carry out its regulatory roles through protein-protein interactions that primarily involve PtsN, although relatively few PtsN protein targets have been characterized. Both phosphostates of PtsN appear active, but the unphosphorylated form predominates among known interactions. Additionally, there are at least a few instances of regulatory roles mediated by PtsP or PtsO separately from their role in phosphotransfer to PtsN, as described in more detail below.

When accepting phosphate from PEP, both PtsP and EI autophosphorylate (4, 9). A key difference between PtsP and EI is that the autophosphorylation activity of PtsP is modulated by its direct interaction with its two ligands, α-ketoglutarate and glutamine. These ligands have opposite effects on PtsP autophosphorylation *in vitro* and on downstream phosphate flow *in vitro* and *in vivo*: glutamine is inhibitory, whereas α-ketoglutarate is stimulatory (9). As mentioned above, the ratio of glutamine to α-ketoglutarate is an indicator of cellular nitrogen availability, with higher glutamine concentrations associated with excess available nitrogen. Interestingly, ligand binding by PtsP requires its GAF domain, which is absent from the EI proteins in PTS^Car^ (3); GAF is a small ligand-binding domain named after the proteins in which it was originally described (cGMP-specific phosphodiesterases, adenylyl cyclases, and FhlA) (17, 18). Thus, ligand binding through the PtsP GAF domain appears to regulate downstream phosphorylation of the other PTS^Ntr^ enzymes (9, 19). Although the GAF-dependent regulation model has been experimentally validated for *E. coli* and at least some other bacteria (9, 10, 19), it has not been validated for PTS^Ntr^ in all bacteria, leaving open the possibility that some systems may function differently from this model.

PtsP, PtsO, and PtsN share varying levels of sequence identity (>20%) with each of their counterparts in the PTS^Car^ (EI, HPr, and EIIA, respectively), but are particularly similar in the vicinity of the phosphorylated histidine (2, 13, 20). The structures of PtsO and PtsN have been solved and compared with those of their counterparts HPr and EIIA (21–26). PtsN has a domain around its active histidine residue that is the site of PtsO interaction, and it makes direct contacts with PtsO, similar to the interactions between EIIA and HPr (21, 24, 25). However, structural studies revealed a key difference between EIIA and PtsN. Whereas EIIA harbors a second conserved histidine within the HPr interaction domain that participates in phosphate transfer to EIIB, in PtsN the equivalent residue faces away from the active site, rendering it unlikely that PtsN participates as a phosphate donor to another protein (2, 22, 26). In support of this idea, no PtsN targets have yet been identified as phosphoacceptors of PtsN.

### Gene distribution and organization

Homologous genes encoding PTS^Ntr^ enzymes are found across all proteobacterial branches except the ε-subdivision, and they are also found in *Chlamydiales*, *Spirochaetales,* and *Planctomycetales* (20). The PTS^Ntr^ is absent in gram-positive species, in contrast to the PTS^Car^ (27). However, the PTS^Ntr^ is the more common of the two PTS in proteobacteria, as the PTS^Car^ enzymes are nearly absent in α- and β-proteobacteria, as well as in *Chlamydiales*, *Spirochaetales,* and *Planctomycetales* (28). Within the δ and γ subdivisions of proteobacteria, most species have both PTS^Ntr^ and PTS^Car^ systems, including *E. coli* and *Pseudomonas aeruginosa* (both γ-proteobacteria) (27). An interesting exception is *Legionella pneumophila* (also γ), which codes for the PTS^Car^ EI and HPr but lacks any recognizable EII or PtsN domains (28), a conclusion supported by co-occurrence in the STRING database of known and predicted protein networks (29). However, STRING analysis indicates variability of these components in other *Legionella* species, with some encoding all the PTS^Ntr^ components and others lacking PtsN, PtsO, or both (29). Other notable genera that lack PtsN include *Acinetobacter* (which encodes PtsP and PtsO) and *Moraxella* (29).

**Fig 2:**
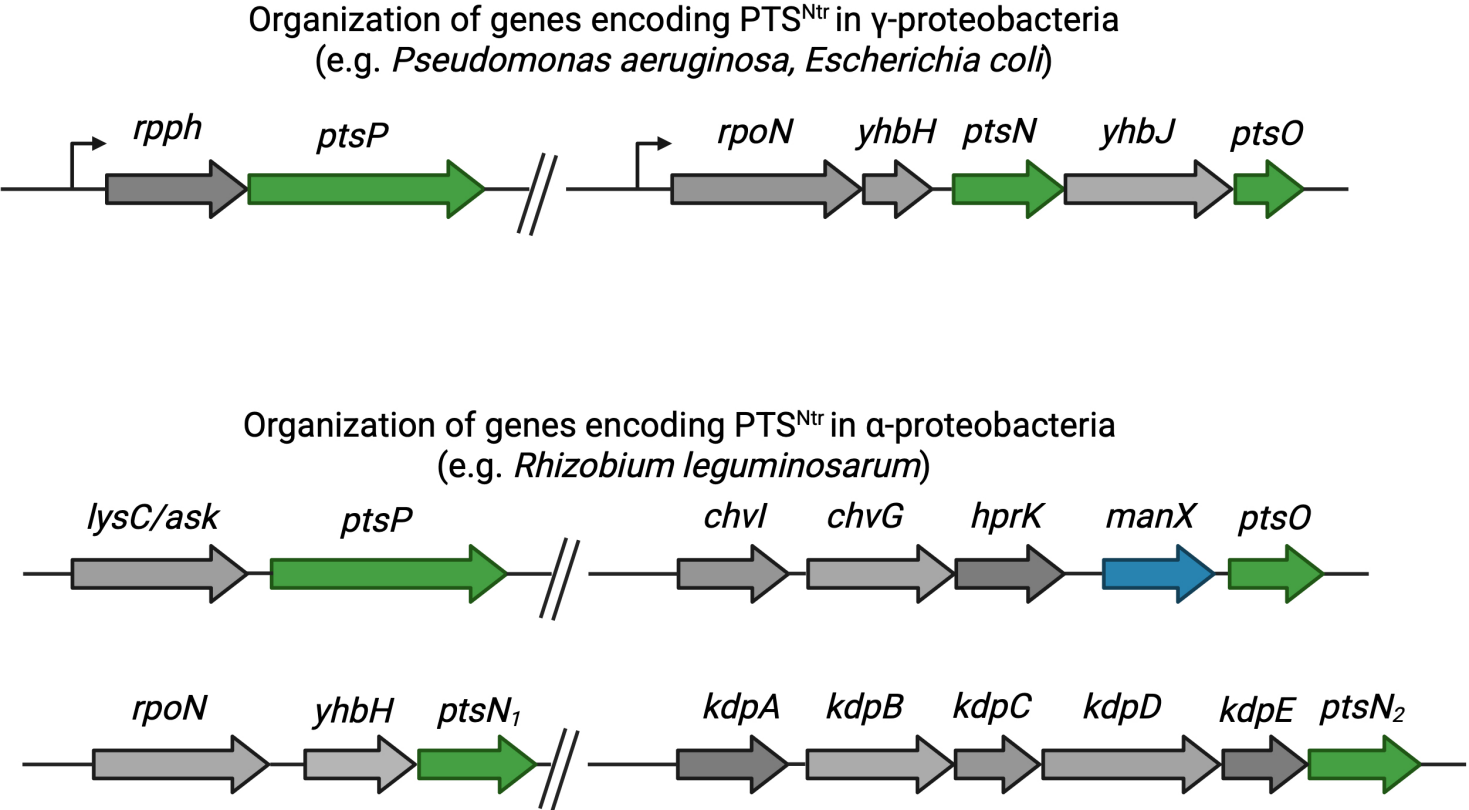
Organization of genes encoding the major components of PTS^Ntr^ in different clades of proteobacteria. Top: gene organization in γ-proteobacteria. Genes of PTS^Ntr^ are shown in green. Bottom: gene organization in α-proteobacteria. Genes encoding PTS^Ntr^ components are shown in green, and blue indicates the *manX* gene, which encodes a PtsO phosphoacceptor that is related to PtsN.

In most δ- and γ-proteobacteria, the PTS^Ntr^ components are encoded in two different operons (Fig. 2A). The *ptsN* and *ptsO* genes are downstream of *rpoN* and are transcribed from the same promoter just upstream of *rpoN* (30) (Fig. 2). Two other genes are also in the *rpoN* operon; *yhbH* is immediately downstream of *rpoN* and encodes the ribosome-protecting hibernation-promoting factor (31), and *yhbJ* (also known as *rapZ*) is between *ptsN* and *ptsO* and post-transcriptionally regulates the glucosamine-6-phosphate synthase GlmS (32, 33). The *ptsP* gene is encoded elsewhere in the genome, typically downstream of the gene *rppH*, which encodes a Nudix RNA pyrophosphohydrolase (formerly named YgdP) (34, 35); this enzyme modifies and promotes the degradation of mRNA of certain gene targets (36, 37). As noted above, some of these gene functions have a role in nitrogen response; for example, *rpoN* encodes σ^54^, which regulates other genes involved in nitrogen response and metabolism (38), and glucosamine-6-phosphate (regulated by the products of *yhbJ/rapZ*) (32) can be used as a nitrogen source.

In α-proteobacteria, the location of *ptsN* within the *rpoN* operon is conserved with other proteobacterial clades, but *ptsO* is in a different operon downstream of *manX*, which codes for a homolog of the mannose-related EIIA terminal phosphoacceptor (Fig. 2B). There are no EIIB or EIIC enzymes in most α-proteobacteria, and it appears that both ManX and PtsN are terminal phosphoacceptors of PtsO (10). Further upstream of *ptsO* and *manX* is *hprK,* which encodes a kinase/phosphatase common to many gram-positive bacteria but absent in most other proteobacteria, including *E. coli* (30). Upstream of *hprK* are the *chvI/chvC* genes, which encode a two-component regulatory system that has been shown to interact directly with PtsN (10). About 10% of α-proteobacterial genomes also have a second copy of *ptsN* that is in an operon with the *kdpABCDE* genes, coding for the high-affinity potassium (K^+^) transport system (20). As described in more detail below, PTS^Ntr^ regulates K^+^ homeostasis in several species, and the location of *ptsN* within this operon supports an important role for PTS^Ntr^ in the regulation of cellular K^+^ levels. As in δ- and γ-proteobacteria, in α-proteobacteria, *ptsP* is in a separate operon from the other PTS^Ntr^ genes but downstream of an aspartokinase encoded by the *lysC* (also called *ask*) gene.

### Crosstalk with other phosphorylation systems

Not surprisingly, in bacteria that have both PTS^Ntr^ and PTS^Car^, crosstalk can occur between these two systems. Some of the most direct evidence of crosstalk comes from studies of *Pseudomonas putida* (39) and *P. aeruginosa* (19), where crosstalk has been demonstrated between the PTS^Ntr^ and the fructose PTS (PTS^Fru^). In *P. putida*, the HPr enzyme (FruB) of the PTS^Fru^ can phosphorylate PtsN, thereby acting as an alternative PtsN phosphodonor; FruB phosphorylates PtsN at the same site as PtsO (39). This crosstalk occurs only during growth on fructose and not with other carbon sources due to fructose-relieved transcriptional repression of the fructose-specific PTS^Fru^ genes; repression is by FruR, a protein in the Cra (catabolite repressor/activator) protein family (39–41).

A similar mechanism of FruB-PtsN-directed crosstalk also occurs in *P. aeruginosa*. In a study by Underhill et al. (19), FruB was shown to phosphorylate PtsN but only in the absence of PtsO. In this case, cross-phosphorylation occurs in cells grown on glycerol and glucose, unlike the situation in *P. putida*. These results suggest that in *P. aeruginosa,* the conditions for FruB activity may have diverged from those in *P. putida*. Further, in *P. aeruginosa,* PtsO appears to function as a specificity factor that prevents FruB-directed phosphorylation of PtsN and thereby modulates crosstalk between systems. Interestingly, in the absence of PtsO, PtsN phosphorylation by PtsP was observed, suggesting that PtsO confers specificity on PtsN by blocking phosphorylation by other enzymes more generally. An alternative explanation is that, when PtsO is present, it removes phosphates from PtsN that were donated by FruB or PtsP. Such a function is supported by the existence of a PtsO phosphatase, SixA (42, 43), that might drain phosphates from PtsO, thereby reversing the direction of phosphate flow (see below).

## REGULATORY ROLES OF THE PTS^Ntr^

### A nitrogen-responsive metabolic “switch”

Studies in several species have supported a role for PTS^Ntr^ in regulating metabolic changes in response to nitrogen availability. As described above, the first direct link between PTS^Ntr^ and nitrogen-dependent regulation was the discovery that the first enzyme of this system, PtsP, directly interacts with glutamine and α-ketoglutarate via its GAF domain, resulting in modulation of its ability to autophosphorylate (9). The ratio of these ligands is a proxy for the availability of nitrogen and carbon in the cell; thus, cellular nitrogen levels, or more specifically the ratio of nitrogen to carbon, direct changes in phosphate flow through the PTS^Ntr^ and therefore regulation of its downstream targets.

**Fig 3:**
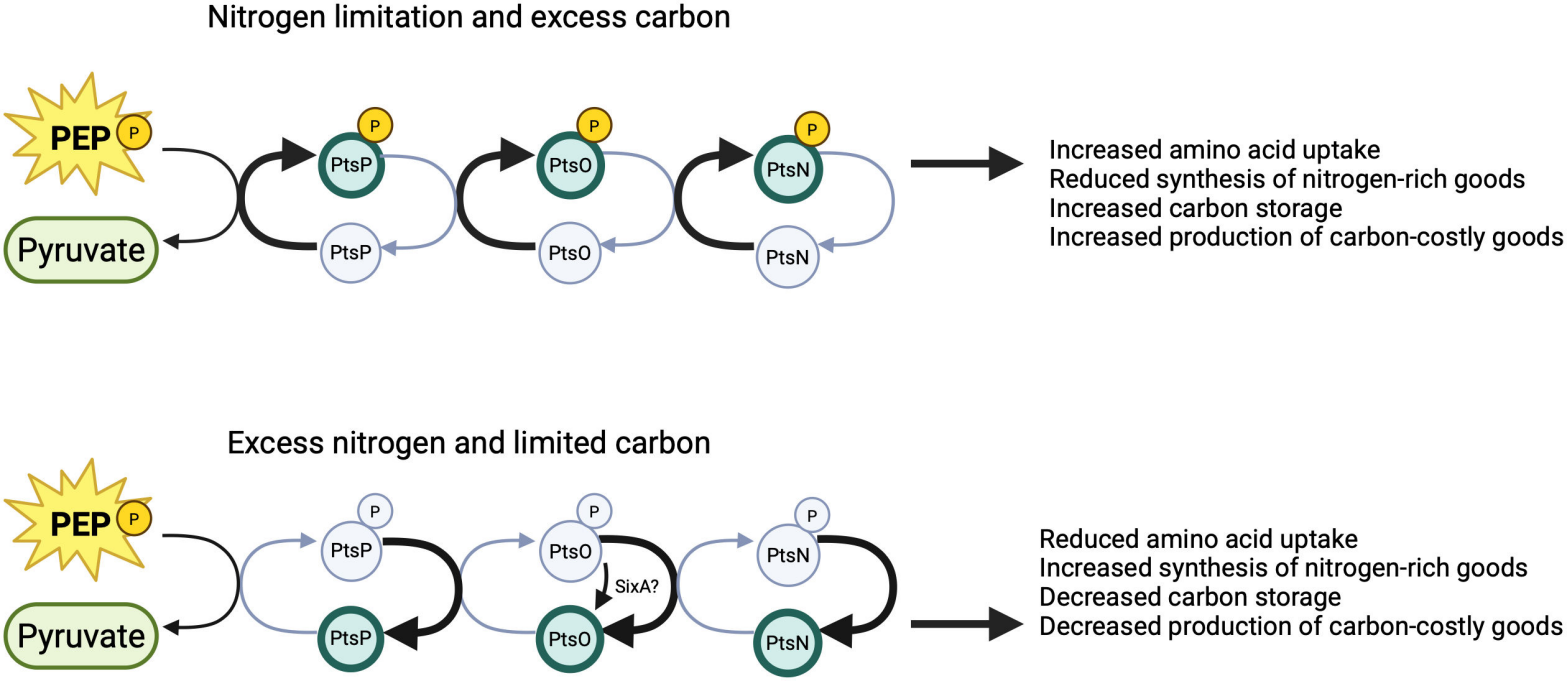
Model of PTS^Ntr^ nitrogen response and related functions. In the presence of excess carbon (more α-ketoglutarate), PtsP autophosphorylation is stimulated, resulting in PtsN being phosphorylated (top panel). Stimulation of phosphotransfer generally results in nitrogen conservation and carbon usage or storage. In the presence of excess nitrogen (more glutamine), PtsP autophosphorylation is inhibited, resulting in PtsN being unphosphorylated (bottom panel). Phosphates may also be taken from the system by the phosphatase SixA. Here, the regulatory outcomes conserve carbon while using nitrogen or limiting its uptake. The phosphorylation status of the PTS^Ntr^ enzymes also modulates potassium homeostasis, virulence, stress responses, and other functions with varying effects in different species as outlined in the text.

The available evidence supports a general model for nitrogen-dependent regulation as depicted in Fig. 3. Under nitrogen-limited and excess-carbon conditions (more α-ketoglutarate), the PTS^Ntr^ enzymes are phosphorylated, which in turn increases amino acid uptake for nitrogen acquisition, redirects carbon flow through central metabolism to prevent nitrogen waste, and shunts carbon into storage and other carbon-costly processes such as the production of exopolysaccharides. Conversely, in nitrogen-rich, carbon-limited conditions (more glutamine), the PTS^Ntr^ enzymes remain unphosphorylated, which in turn downregulates amino acid uptake and instead directs energy and resources into synthesis of nitrogen-containing compounds and carbon catabolism.

A comprehensive analysis of this model was conducted in a recent study by Sánchez-Cañizares et al. in *Rhizobium leguminosarum* (10). This nitrogen-fixing α-proteobacterium lives in symbiosis with the roots of legumes. In *R. leguminosarum*, PTS^Ntr^ involves PtsP, PtsO (more frequently referred to as NPr in this organism), and two phosphoacceptors, PtsN and ManX (Fig. 2, bottom). There is also a second copy of *ptsN* located on a plasmid (44). This study showed that, consistent with the model above, simulating nitrogen-limited conditions by using phosphomimetic versions of ManX and/or PtsN directed downregulation of TCA activity, increased exopolysaccharide production, and increased amino acid uptake. Conversely, blocking phosphate flow to ManX and PtsN or growing cells with an abundance of nitrogen (e.g., glutamine) activated the TCA cycle. This regulation was dependent on the PtsP GAF domain, consistent with the model that ligand-dependent modulation of PtsP drives PTS^Ntr^-dependent regulatory effects. Together, the results support the idea that the PTS^Ntr^ effects important changes in metabolism in *Rhizobium leguminosarum* in response to cellular nitrogen availability.

There is also evidence from studies of other species supporting the model presented in Fig. 3. For example, in *E. coli,* phosphate flow through the PTS^Ntr^ (indicative of limited nitrogen) inhibits biosynthesis of several nitrogen-rich amino acids. The best-understood example of this involves the branched-chain amino acid (BCAA) biosynthesis pathway responsible for synthesizing leucine, valine, and isoleucine. In this case, unphosphorylated PtsN (indicating nitrogen excess) activates transcription of the *ilvBN* genes encoding the first enzyme of the BCAA pathway, the acetohydroxyacid synthase isoenzyme. Thus, inhibiting phosphate flow through PTS^Ntr^ increases BCAA biosynthesis. This finding was connected back to a result from an earlier study (Powell et al., referenced above), showing that *ptsN* gene disruption caused growth delays in the presence of excess leucine (7). It was later proposed that this was due to both ∆*ptsN-*dependent effects on BCAA biosynthesis and leucine-dependent feedback inhibition on the BCAA pathway, which together caused valine and isoleucine pseudoauxotrophy (45, 46). In addition to BCAA, blocking phosphate flow through the PTS^Ntr^ also modulates cellular levels of aromatic amino acids, which are synthesized through a pathway distinct from that of BCAA (47). Both of these are examples where PTS^Ntr^ modulates amino acid biosynthesis, and therefore the utilization of nitrogen, in response to changes in cellular nitrogen.

There is also evidence from other species that PTS^Ntr^ modulates carbon flux. In *Salmonella enterica* Typhimurium, the phosphorylated form of PtsN (indicative of limited nitrogen) directly interacts with and inhibits GlmS (48), which converts fructose-6-phosphate and glutamine into glucosamine-6-phosphate, a building block of many nitrogen-containing cellular components such as exopolysaccharides and peptidoglycan. Thus, when nitrogen is limited, PTS^Ntr^ reduces glutamine consumption by inhibiting GlmS. PTS^Ntr^ was also linked to central metabolism in *E. coli*, where a ligand-fishing experiment with unphosphorylated PtsN uncovered interactions with enzymes of the TCA cycle (succinate dehydrogenase SdhB and succinyl-CoA ligase SucC), the glyoxylate shunt (isocitrate lyase and malate synthase AceA/B), and gluconeogenesis (malic enzyme MaeB and phosphoenolpyruvate synthase PpsA) (47).

Studies of *P. putida* have also contributed to our understanding of the PTS^Ntr^ and nitrogen/carbon-dependent shifts in metabolism. In *P. putida*, the PTS^Ntr^ regulates accumulation of the industrially important compound polyhydroxyalkanoate (49), which is used by bacteria as a means of storing excess carbon. Unphosphorylated PtsN (indicative of excess nitrogen and limited carbon) decreases polyhydroxyalkanoate stores (49), consistent with the above model. The unphosphorylated form of PtsN also directly binds to and inhibits pyruvate dehydrogenase (50), which generates the first TCA substrate, acetyl-CoA, and effectively redirects carbon flow by slowing the TCA cycle (41). Redirection of carbon to other metabolic processes may explain the impact of the PTS^Ntr^ on polyhydroxyalkanoate production.

### Regulation of potassium homeostasis

Another principal function of the PTS^Ntr^ appears to be regulation of potassium (K^+^) homeostasis; this function was also one of the first known roles of these systems. Balancing K^+^ homeostasis through the regulation of its import and export is important for mitigating osmotic stress and intracellular pH and for changing cell volume, which is increasingly becoming appreciated for its role in altering enzyme reaction kinetics and protein-protein interactions (51–54). These changes could, in principle, influence metabolic and other processes in the cell to balance carbon and nitrogen usage, although the role of PTS^Ntr^ in potassium homeostasis could have other benefits that remain unclear.

The connection between the PTS^Ntr^ and K^+^ homeostasis was initially observed in *E. coli*, where the growth of a ∆*ptsN* mutant was found to be inhibited by K^+^ concentrations above 5 mM in the growth medium (55). This K^+^ sensitivity was later shown to be due to the role of PTS^Ntr^ in regulating intracellular K^+^ levels, although the experimental evidence has led to discrepant mechanistic models (55–58). In *E. coli*, unphosphorylated PtsN regulates several K^+^ transporters through direct protein-protein interactions with a K^+^ importer, TrkA (55), an exporter, YcgO (57, 58), and KdpD, a transcriptional regulator of the transport complex KdpFABC (56, 59). Initial work suggested that PtsN-dependent K^+^ sensitivity was due to its interaction with TrkA and KdpD (55, 56, 59, 60), which have complex effects on intracellular K^+^ concentrations but overall resulted in K^+^ hyperaccumulation in Δ*ptsN* strains (55). However, a more recent study by Sharma et al. (58) suggested that the K^+^ sensitivity of a Δ*ptsN* strain was due to intracellular K^+^ limitation rather than accumulation. They showed that ∆*ptsN* strains displayed decreased cellular K^+^ levels, which, along with K^+^ sensitivity, were relieved by deletion of *ygcO*. The model proposes that the absence of PtsN leads to unfettered YgcO-mediated K^+^ efflux by YcgO and that YgcO efflux activity is further stimulated by high extracellular K^+^, thus depriving cells of intracellular K^+^ and leading to a growth defect (57, 58).

PTS^Ntr^-dependent regulation of K^+^ homeostasis is also conserved in other proteobacteria. For example, PtsN regulates the sensor kinase KdpD via direct protein-protein interaction in *P. putida* and the plant root symbionts *R. leguminosarum* and *Sinorhizobium fredii,* as it does in *E. coli* (60, 61). In the case of *P. putida,* the active form of PtsN is phosphorylated (61), whereas in the others, it is the unphosphorylated PtsN that interacts with KdpD (Fig. 2). In the plant root symbionts, there are two *ptsN* genes encoded in the genome, with the one encoded within the *kdpDE* gene cluster referred to as *ptsN_2_* (Fig. 2) (20, 60). These findings support that regulation of K^+^ homeostasis is another unifying theme of PTS^Ntr^ due to the conservation of this regulatory target across multiple species and environments, although the particular regulatory mechanisms appear to somewhat vary. Whether the control of potassium homeostasis influences nitrogen and carbon metabolism through changes in cellular pH or macromolecular crowding (54) or has an entirely different purpose remains unclear.

### Virulence

The PTS^Ntr^ has been implicated in the virulence of plant and animal pathogens in multiple infection models, which is perhaps unsurprising given its central roles in metabolism and potassium homeostasis. For example, in the intracellular pathogen *Legionella pneumophila*, which lacks any EII component, deleting *ptsP* decreases virulence in a guinea pig model (62), while a different study showed that deleting *ptsP* or *ptsO* reduces intracellular growth (63). The PTS^Ntr^ is also important for intracellular replication of *Brucella* (64, 65) by activating a major virulence determinant, type IV secretion (66). In *S. enterica* Typhimurium, the PTS^Ntr^ is key for utilizing 1,2-propanediol, a carbon source produced by other gut bacteria, which provides an advantage over other gut bacteria during intestinal colonization (67). In this case, PtsN enhances glutathione availability, which increases transcription of the *pdu* operon through an unknown mechanism to allow utilization of 1,2-propanediol (67). How PtsN influences glutathione levels is not yet understood, though it evidently does not alter the expression of genes involved in glutathione synthesis and degradation (67).

The role of the PTS^Ntr^ in virulence has also been studied in *P. aeruginosa,* where its effects have been demonstrated in multiple plant and animal infection models. An early study of a transposon-disrupted *ptsP* mutant showed that this mutation severely attenuated virulence in a mouse burn wound infection model, as well as in *Caenorhabditis elegans* and on plant leaves (68). Subsequent studies confirmed the role of *ptsP* in *P. aeruginosa* virulence (69, 70). The mechanism(s) of PtsP-dependent virulence effects have not been clearly elucidated during infections *in vivo*, although PtsP has been implicated in the activation of several major virulence determinants. These include sensitivity to host-derived surfactants (71), swarming motility (70), and biofilm formation (72). Biofilm formation is suppressed by unphosphorylated PtsN (in Δ*ptsP* or Δ*ptsO* strains) (72), although disrupting the phosphorylation site of PtsN does not appear to impact biofilm formation in the same way as a *ptsP* deletion (19), an interesting phenomenon that has also been observed for some phenotypes in *E. coli* (73). Paradoxically, *ptsP* disruption increases production of the virulence factor pyocyanin and stimulates the activity of the virulence-regulatory quorum-sensing system (74, 75). It is possible that these latter regulation effects are irrelevant in an infection environment, as *ptsP* mutations are not common in clinical isolates (76). It is also interesting that, at least in laboratory cultures, disrupting *ptsP* increases resistance to aminoglycoside antibiotics by an as-yet unknown mechanism (74, 77–79), although the potential implications to infections have not been examined. PtsP may also have some broadly conserved virulence-related mechanisms in other Pseudomonads, as disrupting *ptsP* decreases root colonization by *Pseudomonas fluorescens* and decreases its biofilm formation and antimicrobial production (80, 81).

### Stress response

Another theme that unifies the PTS^Ntr^ systems across different species is its role in regulating stress responses. Several species use these systems to regulate the stringent response, a system that is activated in response to nutrient starvation. The stringent response involves the production and response to a small-molecule “alarmone,” (p)ppGpp, whose synthesis relies on the enzymes RelA and SpoT. PTS^Ntr^ seems to exert its effects by regulating SpoT in both *Caulobacter crescentus* (82) and *Ralstonia eutropha* (83). In the latter case, PtsN interacts directly with SpoT1 (83). Studies in *E. coli* have not directly linked the PTS^Ntr^ with alarmone synthesis but have uncovered connections with other types of stress responses. One example is phosphate stress; unphosphorylated PtsN was shown to directly interact with and stimulate PhoR, the histidine kinase of the phosphate starvation-responsive PhoRB two-component system (84), which may indirectly connect to alarmone synthesis (85). The regulation of K^+^ homeostasis, as described above, is another example, as is σ^E^ (73). In addition, unphosphorylated or unphosphorylatable PtsO has been shown to confer hypersensitivity to extracellular stressors such as salt, sucrose, ethanol, and SDS (86).

### Regulatory functions of PtsO and PtsP

A newly emerging theme is that PtsO and PtsP have independent functions distinct from their role as phosphocarrier proteins in the PTS^Ntr^. These proteins have shown independence both in terms of their regulatory functions (outputs) and their own regulation (input). One example of an independent regulatory function is in *E. coli,* where PtsO has been shown to directly interact with the lipid A biosynthesis enzyme LpxD, causing its inhibition and subsequent effects on lipid A biosynthesis (87). In addition, in *P. aeruginosa,* PtsO has been shown to regulate the quorum-sensing signal synthase LasI in a manner that is independent of PtsP or PtsN (88). In this case, it appears that phosphorylated PtsO is the effector, although no protein targets have yet been identified. Transcriptomic studies also hint at independent PtsO-mediated transcriptional effects in the absence of PtsN (88), although the basis for such effects remains unknown. There is also an example of an independent regulatory function for PtsP in *Caulobacter crescentus*, where stringent response regulation occurs through a direct interaction of PtsP with the SpoT (p)ppGpp synthase (82). On the input side, the phosphostate of PtsO can evidently be regulated independently of its phosphodonor PtsP and phosphoacceptor PtsN. In *E. coli,* PtsO can be dephosphorylated by SixA (42, 43), a phosphohistidine phosphatase that is well conserved across all types of bacteria (89). In addition to PtsO, SixA is a phosphatase of the anaerobiosis regulator ArcB (89), which was its first known function. Thus, SixA can serve as an additional input for PTS^Ntr^ through its modulation of the PtsO phosphorylation state. By draining phosphates from PtsO, SixA could also influence PtsN phosphorylation through its effects on PtsO; in principle, dephosphorylated PtsO could remove phosphates from PtsN, resulting in a lower level of phosphorylated PtsN in cells. The potential regulatory role of SixA through its effects on PTS^Ntr^ is not yet well understood.

## DISCUSSION

Over the past several decades, investigations in a wide range of bacterial species have revealed that nitrogen-related phosphotransferase systems carry out an impressive array of functions—from metabolic regulation to virulence, potassium homeostasis, and stress responses. It remains an open question whether these regulatory effects are coordinated to achieve an overall cellular effect or response, or if there are simply many discrete functions. However, evidence is accumulating that the different functions of this system are tied to the ratio of carbon and nitrogen availability. Thus, the complexity of the PTS^Ntr^, with its multiple inputs and outputs, may be directed toward generating a nuanced response to nutrient availability for optimized growth in different conditions. Importantly, PTS^Ntr^ systems are not only structurally conserved across Proteobacteria but are also more broadly distributed in this phylum than their better-known carbohydrate-related counterparts. The prevalence of PTS^Ntr^ systems and their regulation of processes with direct implications for human health, agriculture, and biotechnology—such as central metabolism, virulence, and production of biotechnologically relevant products—makes them a compelling target for deeper investigation. One important distinction to make in future studies is to clarify any roles of PtsP and PtsO that are separate from their phosphotransfer function. In many cases, it is not known whether a phenotype caused by *ptsP* or *ptsO* mutation or deletion is due to shifts in the phosphostate of PtsN or whether the absence of PtsP or PtsO has separate effects. Moving forward, several key questions remain. What is the full spectrum of upstream signals and downstream targets of PTS^Ntr^ in different bacteria? How do the different impacts of the PTS^Ntr^ observed in different species fit together? How do these systems modulate host interactions and microbial fitness in complex environments? And how can these pathways be harnessed or disrupted for therapeutic or biotechnological applications? By consolidating our understanding of conserved mechanisms and species-specific variations among examples of the PTS^Ntr^, we can begin to approach these questions with new clarity and precision, unlocking the potential of this ancient signaling system as both a model of bacterial regulation and a gateway to practical applications.
